# Targeting Melanoma-Associated Fibroblasts (MAFs) with Activated γδ (Vδ2) T Cells: An In Vitro Cytotoxicity Model

**DOI:** 10.3390/ijms241612893

**Published:** 2023-08-17

**Authors:** Anna Hajdara, Uğur Çakır, Barbara Érsek, Pálma Silló, Balázs Széky, Gábor Barna, Shaaban Faqi, Miklós Gyöngy, Sarolta Kárpáti, Krisztián Németh, Balázs Mayer

**Affiliations:** 1 Department of Dermatology, Venereology and Dermatooncology, Semmelweis University, 1085 Budapest, Hungary; hajdara.anna@itk.ppke.hu (A.H.); cakir.ugur@semmelweis-univ.hu (U.Ç.); sillo.palma@med.semmelweis-univ.hu (P.S.); szeky.balazs@itk.ppke.hu (B.S.); shaaban.faqi@phd.semmelweis.hu (S.F.); karpati.sarolta@med.semmelweis-univ.hu (S.K.); nemeth.krisztian@med.semmelweis-univ.hu (K.N.); 2 Roska Tamás Doctoral School of Sciences and Technology, Faculty of Information Technology and Bionics, Pázmány Péter Catholic University, 1083 Budapest, Hungary; 3 Department of Genetics, Cell and Immunobiology, Semmelweis University, 1089 Budapest, Hungary; ersek.barbara@semmelweis.hu; 4 Department of Pathology and Experimental Cancer Research, Semmelweis University, 1085 Budapest, Hungary; barna.gabor@med.semmelweis-univ.hu; 5 Károly Rácz Doctoral School of Clinical Medicine, Semmelweis University, 1085 Budapest, Hungary; 6 Faculty of Information Technology and Bionics, Pázmány Péter Catholic University, 1083 Budapest, Hungary; gyongy.miklos@itk.ppke.hu

**Keywords:** γδ T cells, melanoma-associated fibroblasts, zoledronic acid, melanoma, tumor microenvironment, cancer, cancer-associated fibroblasts, butyrophilin

## Abstract

The tumor microenvironment (TME) has gained considerable scientific attention by playing a role in immunosuppression and tumorigenesis. Besides tumor cells, TME is composed of various other cell types, including cancer-associated fibroblasts (CAFs or MAFs when referring to melanoma-derived CAFs) and tumor-infiltrating lymphocytes (TILs), a subpopulation of which is labeled as γδ T cells. Since the current anti-cancer therapies using γδ T cells in various cancers have exhibited mixed treatment responses, to better understand the γδ T cell biology in melanoma, our research group aimed to investigate whether activated γδ T cells are capable of killing MAFs. To answer this question, we set up an in vitro platform using freshly isolated Vδ2-type γδ T cells and cultured MAFs that were biobanked from our melanoma patients. This study proved that the addition of zoledronic acid (1–2.5 µM) to the γδ T cells was necessary to drive MAFs into apoptosis. The MAF cytotoxicity of γδ T cells was further enhanced by using the stimulatory clone 20.1 of anti-BTN3A1 antibody but was reduced when anti-TCR γδ or anti-BTN2A1 antibodies were used. Since the administration of zoledronic acid is safe and tolerable in humans, our results provide further data for future clinical studies on the treatment of melanoma.

## 1. Introduction

Over recent decades, stromal cells of the TME have emerged as some of the crucial determinants of tumor progression and unfavorable clinical outcomes due to their roles in immune suppression, the development of distant metastases, and local therapy resistance [1,2,3]. The TME is a complex system composed of various cells, including tumor-infiltrating immune cells, CAFs, and endothelial cells embedded in the extracellular matrix. The TME interacts closely with the tumor cells, favoring tumorigenesis [4].

Melanoma is a highly aggressive malignancy of melanocytes, a pigment-producing type of cell [5]. It is among the most immunogenic tumor types and has greater potential to respond to immunotherapy [6]. On the other hand, like other cancer types, melanoma tumor cells acquire various suppressive properties to escape innate and adaptive immune recognition and destruction [6].

MAFs are a melanoma-driven subpopulation of CAFs that promote tumorigenesis through the facilitation of immune evasion and proliferation of cancer cells [7,8]. Their exact origin in the TME is under debate. Several studies support the notion that they originate from a group of activated local fibroblasts in the tissue [9,10]. Other studies reported that melanoma-derived exosomes can promote the differentiation of endothelial cells into CAFs through the endothelial–mesenchymal transition (EMT). In the skin, the activation of fibroblasts might lead to the recruitment of stromal cells by tumor cells through paracrine signaling due to the pro-tumorigenic milieu and the secretory activity of melanoma cells [7].

In the TME, tumor-infiltrating immune cells contain a unique population of immune cells, γδ T cells, which are a subset of T lymphocytes that carry the immunological features of the innate and adaptive immune system [11]. Generally, they are divided into two major structural subsets (note that other minor subsets also exist) based on their T cell receptor (TCR) δ chain form: Vδ1 and Vδ2 [12]. Vδ1 cells are predominantly associated with the Vγ1 gene family (Vγ2/3/4/5/8), while Vδ2 T cells co-express VγII (Vγ9) [13]. Vδ2 cells are primarily located in the peripheral blood, whereas Vδ1 cells reside in the gut epithelia, dermis, spleen, and liver and contribute to the maintenance of epithelial tissue integrity [11]. In contrast with the conventional T cells that bear the αβ T cell receptor, which recognizes antigen-driven peptides through their major histocompatibility complex (MHC) cell surface molecules (human leukocyte antigen in humans), the ligand recognition of γδ T cells is not MHC-restricted [14]. Their activation is dependent on non-peptidic pyrophosphate antigens (P-Ags), such as isopentenyl pyrophosphate (IPP), a product of the mevalonate pathway that is used by eukaryotic cells and recognized by the Vγ9/Vδ2 T cell receptor [15]. Pharmacological inhibitors of IPP catabolism, such as aminobisphosphonates, are able to sensitize the target cell recognition of Vγ9/Vδ2 T cells, thus causing the accumulation of IPP in the target cell [16,17]. In addition, IPPs are also upregulated in metabolic disorders [18] and in malignantly transformed cells that are prone to undergoing metabolic disturbance. γδ T cell accumulation in cancer tissues can also be the best predictor of better prognosis among 22 different types of immune cells across 39 malignancies [19].

Their MHC-independent ligand recognition and their unique mode of activation directed our attention to γδ T cells. Both Vδ1 and Vδ2 T cells have been found in melanoma tumor tissue. Vδ2 T cells could be isolated from the peripheral blood of melanoma patients, and these cells could still be expanded but had a reduced cytotoxic activity against tumor cells ex vivo [20,21]. Current therapies using γδ T cells to target solid tumors are not efficient enough, and new treatment modalities are needed [22].

Recently, our research group studied MAFs and showed that these cells suppress cytotoxic T lymphocyte activity [23]. We also found that, similarly to bone marrow mesenchymal stromal cells (BMSCs or MSCs), MAFs drive macrophages to produce IL-10, an anti-inflammatory cytokine that suppresses natural anti-cancer immunity [24].

Considering the immunosuppressive TME and to address recent challenges of γδ T cell therapies, we focused on MAFs instead of tumor cells [22]. We set up a new apoptosis model to separately study blood-derived Vγ9/Vδ2 T cells and MAF interactions in vitro and to answer the question of whether it is possible to kill MAFs with activated Vδ2 T cells (referred to from here on as γδ T cells).

For the activation of γδ T cells, we used zoledronic acid (ZA; also known as zoledronate), which represents a newer generation of aminobisphosphonate drugs [25]. ZA is used to prevent bone complications in adults in advanced cancer and to treat hypercalcemia caused by tumors. ZA stops the action of osteoclasts and reduces bone loss and the amount of calcium released into the blood [25].

## 2. Results

### 2.1. Peripheral γδ T Cells Can Be Expanded In Vitro via Zoledronic Acid (ZA) Stimulation

**Figure 1:**
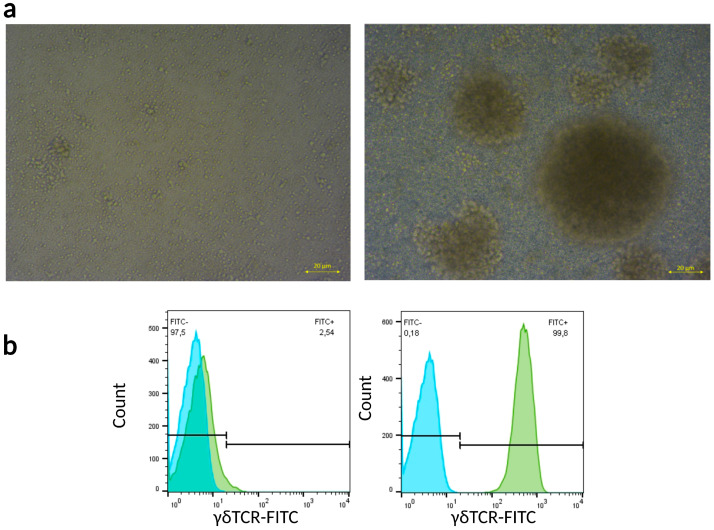
ZA and IL-2 mediated PBMC proliferation and magnetic separation of γδ T cells. (**a**) Morphology of unstimulated (**left**) and ZA- and IL-2-stimulated PBMCs 7 days post-stimulus (**right**). Scale bar = 20 µm. (**b**) Representative flow cytometry histograms of γδTCR-negative (**left**) and -positive (**right**) PBMC fractions after magnetic separation. Blue: unlabeled cells, green: γδTCR-FITC-labeled cells. Separation purity (n = 4): 99.2 ± 0.43.

As the proportion of γδ T cells among peripheral blood mononuclear cells (PBMCs) is between 1 and 5% [26], we expanded γδ T cells for our in vitro experiments by stimulating them with ZA according to a published protocol [27]. After the third day of ZA stimulus, a typical cell aggregate formation was observed as the activated γδ T cells started to proliferate (Figure 1a). On day 7, PBMCs were magnetically separated, and two fractions of the separation process were analyzed via flow cytometry. The magnetically retained-then-eluted fraction contained a homogeneous population of γδ T cells (the purity was approximately 99%) at each separation throughout our experiments (Figure 1b).

### 2.2. Flow Cytometry Apoptosis Assays in γδ T Cell–MAF Co-Cultures

**Figure 2:**
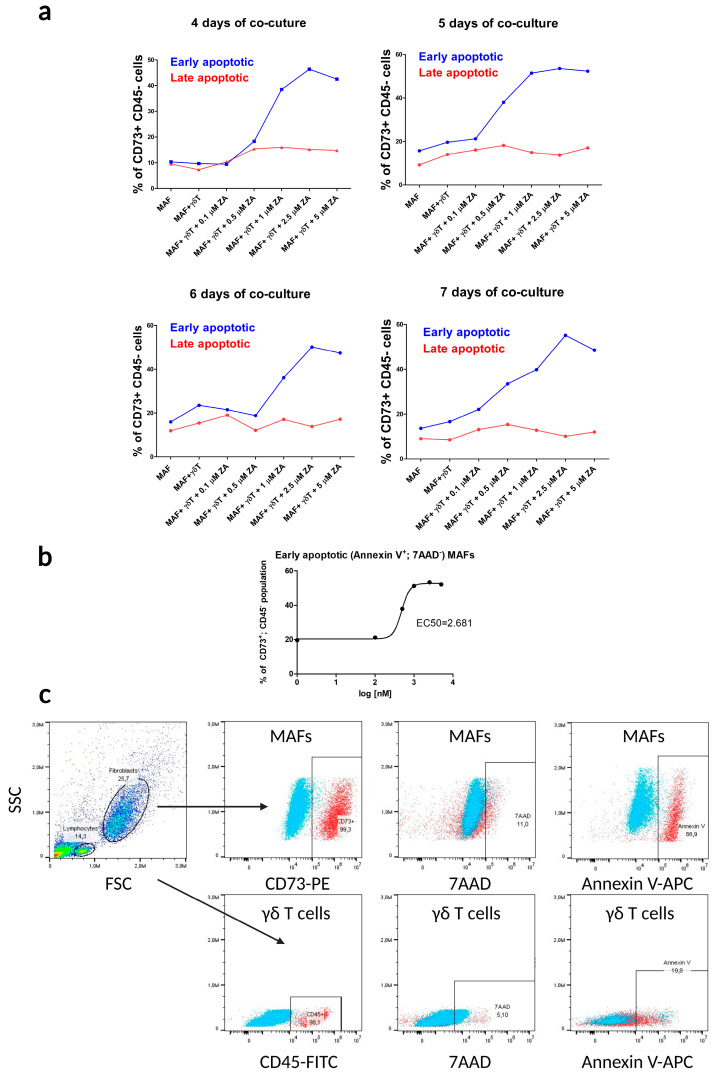
Optimization of flow cytometry apoptosis assays in γδ T cell–MAF co-cultures. γδ T cell–MAF co-cultures were stimulated with ZA, and early (Annexin V+^,^ 7AAD−) and late (Annexin V+ 7AAD+) apoptotic cell populations were measured. (**a**) Concentration-dependent effect of ZA on MAF apoptosis after various days (4–7) in co-culture with γδ T cells. (**b**) The EC50 value of ZA was determined for the early apoptotic MAFs (CD73+, CD45−, Annexin V+, 7AAD−) in the co-culture on the fifth day. (**c**) Gating strategy of flow cytometry analysis: determining main cell populations according to their side scatter and their CD73+ and CD45+ surface markers. Lastly, Annexin V and 7AAD-positive cells within the CD73+ and CD45+ cell populations were analyzed (blue: unlabeled control cells).

First, we set up our co-culture apoptosis assay to test different timings (4–7 days) and different ZA concentrations (Figure 2a). We found that 5 days of co-culture and 1 and 2.5 µM ZA stimulations were the optimal conditions for our assay. Using flow cytometry and a CCK-8 viability assay, we also examined whether the different concentrations of ZA affected the apoptosis and viability of MAFs in mono-culture without γδ T cells (Supplementary Figure S1). There was no significant increase in apoptosis or decrease in viability of MAFs treated with different concentrations of ZA (0–5 µM) for 5 days (see Supplementary Figure S1a,b and the Supplementary Methods).

We also investigated whether pre-stimulation of γδ T cells (7 days of pre-stimulation of PBMCs during γδ T cell enrichment by using ZA and IL-2 prior to the 5-day co-culture assay) was sufficient to exert their cytotoxic effects on MAFs. We found that there was no significant difference in the co-culture of non-stimulated γδ T cells with MAFs and the MAF mono-culture in terms of early apoptosis of MAFs, thus supporting the finding that pre-stimulated γδ T cells (Figure 2a) need further activation by ZA in co-culture with MAFs to enhance their cytotoxic activity.

We compared early and late apoptotic MAF populations (percentages). The apoptotic populations of the MAF mono-culture, the non-stimulated co-culture, and the lowest 0.1 μM ZA-stimulated γδ T cell-MAF co-culture showed minimal differences in the early and late apoptotic populations. By increasing the ZA concentration, the early apoptotic populations also increased, while the late apoptotic one remained below 20% (Figure 2a).

**Figure 3:**
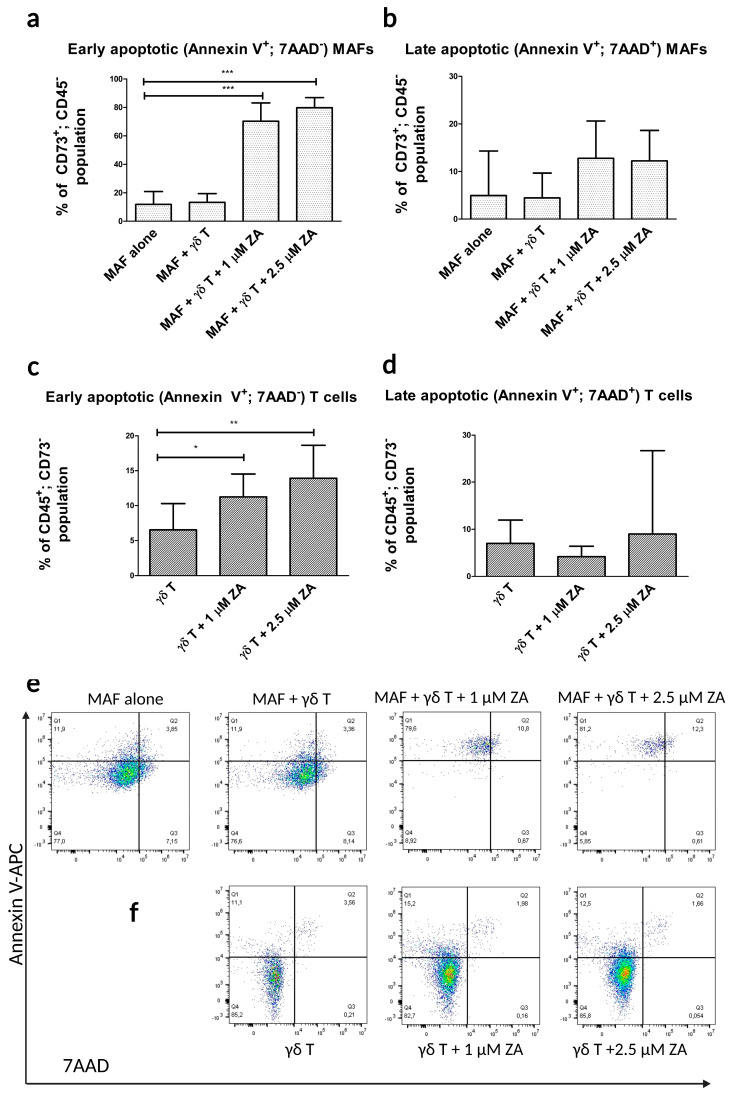
Flow cytometry apoptosis assays in γδ T cell and MAF co-cultures using the formerly optimized conditions (5 days of co-culture with 1 μM and 2.5 μM ZA-stimulated cells. (**a**) Percentages of early apoptotic (Annexin V+, 7AAD−) and (**b**) late apoptotic (Annexin V+, 7AAD+) populations of MAFs (CD73+; CD45−). n = 10. Control: MAF mono-culture without γδ T cells. (**c**) Percentages of early apoptotic and (**d**) late apoptotic populations of γδ T cells (CD45+; CD73-) after 5 days of co-culture with MAFs. Control: co-culture with unstimulated γδ T cells. (**e**) Flow-cytometric dot plots of apoptotic fibroblast populations. (**f**) Flow-cytometric dot plots of apoptotic γδ T cells. Error bars represent means ± SD. * *p* < 0.05 ** *p* < 0.01 and *** *p* < 0.001.

By using the optimal conditions, we performed our co-culture apoptosis assay with healthy γδ T cells that were stimulated with ZA and with MAFs from different donors. By analyzing MAFs from the co-culture, we found over 60% of the cells were early apoptotic (Figure 3a) and around 10% of the cells were late apoptotic (Figure 3b).

From the co-culture, we also measured early (Figure 3c) and late apoptotic (Figure 3d) populations of γδ T cells, both of which were below 10%. The ZA-stimulated cultures had significantly more early apoptotic γδ T cells than the unstimulated controls (Figure 3c).

### 2.3. MHC Class I Polypeptide-Related Sequences A and B and BTN2A1 and BTN3A1 Molecules Are Expressed in MAFs and in Normal Dermal Fibroblasts (NDFs)

**Figure 4:**
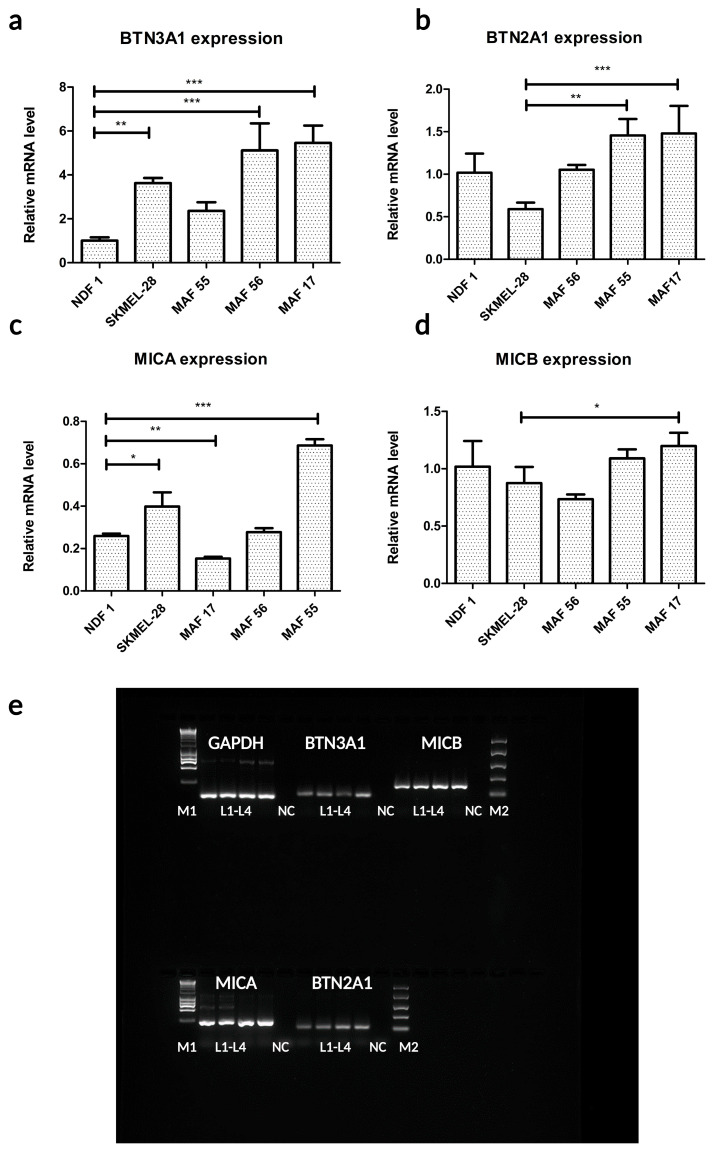
mRNA expression of γδ TCR target molecules in MAFs. Relative mRNA expression of (**a**) BTN3A1, (**b**) BTN2A1, (**c**) MICA, and (**d**) MICB molecules compared to the mRNA expression of the housekeeping gene GAPDH and the mRNA expression of NDFs as a calibrator. SK-MEL-28 melanoma cells were used as positive controls. (**e**) Gel electrophoresis images of the target molecules' RT-PCR products (in order: GAPDH, BTN3A1, MICB, MICA, and BTN2A1). Lanes: M1: marker, 1 kB HyperLadder™; L1–L2: MAF; L3: NDF; L4: SK-MEL-28 positive control; NC: NTC-non template control; M2: marker, 100–2000 bp Easy Ladder. Error bars represent means ± SD. * *p* < 0.05 ** *p* < 0.01 and *** *p* < 0.001.

To explore the molecular details of the cytotoxic effect of γδ T cells on MAFs, we analyzed the gene expression of the main γδ T cell receptor (γδ TCR and NKG2D) target molecules in MAFs via reverse transcription PCR and quantitative PCR (Figure 4a–d).

The mRNAs of the γδ TCR targets BTN2A1 and BTN3A1 and the NKG2D targets MHC class I polypeptide-related sequences A and B (MICA) and (MICB) were shown to be expressed in MAFs and in NDFs (Figure 4a–d).

The relative increase in BTN3A1 mRNA and the protein level in MAFs (Figure 4a) compared to that in NDFs was also evident (Supplementary Figure S2), which prompted us to study the γδ TCR-BTN axis in greater depth.

### 2.4. γδ-T-Cell-Induced Apoptosis in MAFs Is MHC Independent and Relies on the γδ TCR-Butyrophilin-Axis

**Figure 5:**
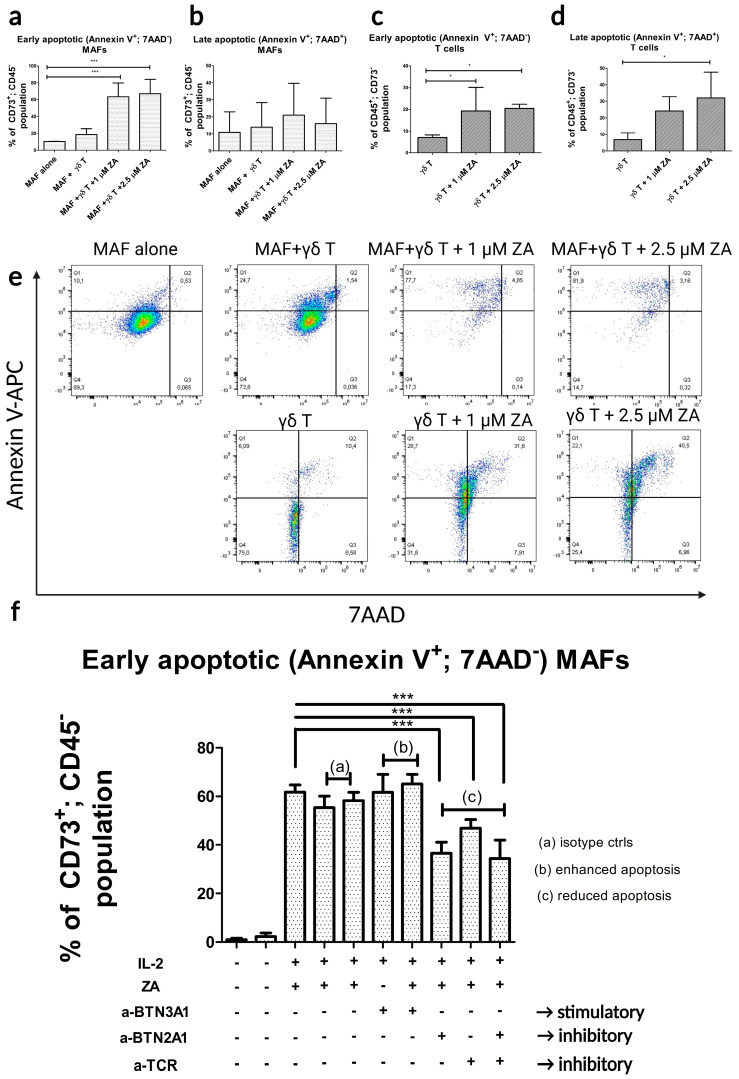
γδ T cell and MAF interaction followed by early (Annexin V+; 7AAD−) and late (Annexin V+ 7AAD+) apoptotic populations. (**a**,**b**) Early and late apoptotic populations of MAFs. (**c**,**d**) Early and late apoptotic populations of γδ T cells. In this experimental setup, both MAFs and γδ T cells were isolated from the same melanoma patients. (**e**) Representative flow-cytometric dot plots of apoptotic MAFs and γδ T cells of the two melanoma patients. (**f**) Early apoptotic populations of MAFs with additional conditions, including BTN2A1 inhibition by anti-BTN2A1 antibody, γδ T cell receptor blocking with anti-TCR γδ (Clone B1) antibody, and BTN3A1 stimulation by CD277 monoclonal antibody (clone 20.1) and mouse IgG isotype control. Error bars represent means ± SD. * *p* < 0.05 *** *p* < 0.001.

Next, we asked whether the cytotoxic effect of γδ T cells on MAFs is MHC-dependent. To answer this question, we repeated the five-day apoptosis assay with MAFs and γδ T cells by matching two previously isolated MAFs with γδ T cells derived from the same donors as those of the MAFs. The γδ T cells without stimulus did not induce apoptosis, although stimulation of the co-culture with ZA and IL-2, a significant increase in early apoptotic MAF population was detected (Figure 5a).

Both the 1 μM and 2.5 μM ZA and IL-2 stimuli of the co-culture resulted in significant increases in the early apoptotic MAF population (Figure 5a). The late apoptotic MAF populations showed no significant differences (Figure 5b). With this setting, we demonstrated that γδ T cells are capable of killing MAF cells bearing the same MHC molecules, so their action was MHC-independent.

Compared to the assays with γδ T cells derived from healthy individuals (Figure 3), the ratio of early apoptotic γδ T cell population was not significantly different (although the mean was a lower percentage). Interestingly, there was significant difference in the late apoptotic γδ T cell population, which increased up to 30% (Figure 5d) compared to the late apoptotic population of healthy γδ T cells (Figure 3d). During the PBMC proliferation in the two melanoma patients, we observed that upon stimulation with ZA, the yield of γδ T cells was lower than that from healthy PBMCs.

To investigate the molecular mechanism of the interaction of γδ T cells and MAFs, the initial apoptosis assay was modified. We applied a special stimulatory clone of anti-BTN3A1 (Clone 20.1) antibody (that enhanced the cytotoxicity of γδ T cells) [28,29,30] at a concentration of 1 μg/mL with or without 1 μM ZA to investigate whether it indeed resulted in an increased apoptotic MAF population. Compared with non-stimulated γδ T cells, both the stimulatory anti-BTN3A1 antibody and the stimulatory anti-BTN3A1 antibody plus ZA resulted in significantly increased early apoptotic (Figure 5f) MAF populations. The dual effect of ZA and the stimulatory anti-BTN3A1 antibody on MAF apoptosis was not significantly different from that of ZA alone.

To demonstrate the direct interaction between γδ T cells and MAFs, we applied 5–5 μg/mL anti-BTN2A1 [29] and anti-TCR γδ (Clone B1) [31] and compared the apoptotic populations with the 1 μM ZA-stimulated co-culture as a control.

We found that the pre-incubations of MAFs with the anti-BTN2A1 antibody and γδ T cells with the anti-TCR γδ antibody significantly decreased the early (Figure 5f) apoptotic MAF populations in our co-culture assay. This inhibition of MAF apoptosis was partial, indicating that other molecules could also be involved in the interaction of γδ T cells and MAFs.

## 3. Discussion

ZA has been given to various cancer patients [32,33] and has been successfully used in the case of a melanoma patient with bone metastasis [34]. Following intravenous zoledronic acid (4 mg) administration, regression of pulmonary and bony metastases was observed, and activation of the patient’s own γδ T cells in the background was hypothesized [28,35,36].

Nevertheless, current therapies using γδ T cells are not efficient enough and still need improvement. Several clinical trials utilizing γδ T cells to target solid tumor cells were withdrawn or terminated (e.g., NCT01606358, NCT05628545, NCT01404702, NCT00582790). As a different approach, we decided to study the apoptosis-inducing effect of γδ T cells on MAFs instead of cancer cells. To answer this question, we set up an in vitro model to separately investigate the interactions of selectively isolated γδ T cells and MAFs. For MAF isolation and culturing, we used our previously published method and excluded MAF cell cultures that were contaminated with melanoma cells. For γδ T cells, we adapted a published protocol and performed control experiments by using MHC-identical MAFs and γδ T cells to prove that the cytotoxicity was MHC-independent and not the result of an unexpected MHC-dependent cytotoxic T cell action.

We hypothesized that the same molecules implicated in γδ T cell–tumor cell interactions may play a role in the cytotoxic activity of γδ T cells on MAFs [37,38]. More specifically, ZA may cause the accumulation of IPP within MAFs. The accumulated IPP molecules bind to the intracellular part of BTN3A1 (and not to that of BTN2A1). Upon IPP binding, BTN3A1 molecules change their conformation and connect with BTN2A1 molecules through their intracellular B30.2 domains. The active conformation enables the extracellular part of BTN2A1 to directly bind to the Vγ9 chain (between the CDR2 and CDR3 regions) of Vγ9/Vδ2 TCR. Once γδ TCR is bound to BTN2A, the cytotoxic mechanism is activated, and the γδ T cell kills the target MAF cells. Note that the binding partner of BTN3A1 on the γδ T cell is not known, but a putative CDR3 ligand that links BTN3A1 and γδ TCR has been hypothesized [39].

In our experiments, we were able to increase the apoptosis of MAFs by using ZA and the BTN3A1-activating antibody clone anti-BTN3A1 20.1, indicating the presence of BTN3A1 proteins on the MAFs’ surface. The apoptosis of MAFs decreased by using the anti-TCR γδ and anti-BTN2A1 antibodies, indicating the direct involvement of these two molecules in the cytotoxic activity of γδ T cells. The limitation of our study is that instead of specific knock-out cells or anti-BTN3A1 103.2 or anti-BTN2A1 7.48 monoclonals, we used NDFs with very low expression of BTN3A1 (see Supplementary Figure S2) and a polyclonal anti-BTN2A1. However, based on our data, we conclude that the TCR γδ–BTN2A1-BTN3A1 axis is a main factor in the apoptotic process.

Partial inhibition of apoptosis in MAFs may mean that other molecules—such as the NKG2D receptors on γδ T cells and their MICA/B targets on MAFs—could also be involved.

Based on the results in our γδ T cell–MAF co-culture cytotoxicity model, we found that pre-activation of γδ T cells during enrichment was not sufficient for their optimal cytotoxic activity. Further activation (ZA or stimulating anti-BTN3A1 clone) is needed to enable their optimal cytotoxic activity, and this activity has its limitations (see Figure 2b: sigmoid curve—plateau phase).

In our experiments, a 1:1 effector–target (E:T) cell ratio was chosen, and this ratio enabled γδ T cells to effectively drive MAFs (approximately 60%; see Figure 3a) into apoptosis when ZA was also present. In other studies, the E:T ratio of 1:1 was effective in long-term assays and through additional stimulation (e.g., ZA) of the co-culture. The cytotoxic effect of γδ T cells could be enhanced in vitro by increasing the E:T ratio (in the literature, up to 10:1 or 50:1) However, we believe that an in vitro E:T ratio of 1:1 is more relevant for an in vivo situation than higher ratios and may be more informative for later in vivo or clinical studies. In this context, in the design of future in vivo experiments, it is important to consider not only the number of MAFs, but also all BTN3A1- and BTN2A1-co-expressing cells that could be potential targets of γδ T cells. We speculate that these might not be only tumor cells and MAFs, but possibly also other immunosuppressive cells of the TME, such as myeloid-derived suppressor cells or tumor-associated macrophages [40,41,42].

We found that a ZA concentration of at least 1–2.5 μM was needed in our assay to allow γδ T cells to drive MAFs into apoptosis. Patients normally receive 4–5 mg of ZA as an intravenous injection (in 100 mL of vehicle, administered over 15 min), which corresponds to a C_max_ of 370 or 471 ng/mL. These maximum concentrations are 1.36 or 1.73 μM, respectively, which approximate the concentrations in our in vitro assay. In the clinical setting, a sustained high intratumoral ZA concentration may be needed, which could be achieved through intralesional administration of ZA or prolonged infusion of ZA if tolerated.

For the optimal cytotoxic effect of γδ T cells, BTN2A1 and BTN3A1 expression and presence on the target cells (MAFs) are necessary. Current studies have shown that BTN2A1 expression on target (cancer) cells is correlated with γδ T cell cytotoxicity [30,43]. Other researchers analyzed Gene Expression Profiling Interactive Analysis (GEPIA) datasets (see also www.gepia.cancer-pku-cn (accessed on 5 July 2023) Copyright © 2017 Zefang Tang, Chenwei Li, Boxi Kang. Zhang’s Lab) [44] and found that higher BTN3A1 expression was associated with longer overall survival in cutaneous melanoma, and increased expression of BTN3A1 was also positively associated with the infiltration of γδ T cells into tumor tissues (lung and breast cancer) [45]. On the other hand, Payne and colleagues reported that, in its spontaneous conformation without BTN2A1, BTN3A1 inhibited tumor-reactive αβ T cell activation. BTN3A1-stimulating antibodies or IPP transformed the immunosuppressive molecule conformation of BTN3A1 into an immunostimulatory one and elicited coordinated restoration of αβ T cell effector activity and BTN2A1-dependent γδ T lymphocyte cytotoxicity against BTN3A1+ cancer cells [46]. Modified expression of molecules belonging to the BTN/BTNL family was reported in clinical studies when an immune checkpoint blockade was applied with PD-1 antibodies, suggesting a role in tumor immune escape [47,48,49]. In addition, in a soluble form, the plasma level of BTN3A1 was correlated with other immune-evasion-favoring molecules in pancreatic cancer patients according to Bian et al. [48] and was negatively correlated with overall survival.

In our quantitative RT-PCR measurements, we did not have enough donors to assess whether BTN2A1 or BTN3A1 expression in MAFs was positively correlated with γδ T cell cytotoxic activity. We think that for an optimal cytotoxic effect, the immunostimulatory conformation of BTN3A1 on MAFs may be more important than its expression level.

Regarding the apoptosis of healthy and patient-derived γδ T cells, we can only speculate that the apoptosis of γδ T cells may be connected with their cytotoxic activity. As the apoptosis of bone marrow stromal cells (mesenchymal stem cells) was described as necessary for immunosuppression [50], one can believe that, as MAFs are similar in their characteristics to mesenchymal stem cells, dying MAFs exhibit an immunosuppressive phenotype [50]. This phenomenon might contribute to the immunosuppressive milieu of the TME and might affect the anti-tumor activity of γδ T cells. Further studies are necessary to investigate this theory.

Recently developed γδ T cell therapies use the stimulatory clones of BTN3A antibodies; as immune checkpoint molecules are expressed on γδ T cells [51], immune checkpoint inhibitors could be an additional treatment for further activating γδ T cells (see trial NCT04243499; BTN3A and PD-1 antibodies). Other new strategies that use bispecific antibodies that are specific for an antigen expressed on cancer cells and for a second target, an activating molecule on γδ T cells (HER2/Vγ9), are on the way. Another novel strategy is chimeric antigen receptor (CAR)-carrying γδ T cells (CAR-γδ T cells) that recognize tumor epitopes independently of their TCR-s.

Finally, we believe that our in vitro data contribute to the accomplishment of future in vivo studies for improving therapies that use ZA-activated γδ T cells alone or in combination with systemic or local ZA injections and stimulating anti-BTN3A1 antibodies or immune-checkpoint inhibitors. In addition, a combination of these strategies with the novel CAR-γδ T cells may be the direction of future studies for overcoming the immunosuppressive TME of melanoma.

## 4. Materials and Methods

### 4.1. Human Samples

Skin biopsies of eleven melanoma patients (clinical data of the patients can be found in Table 1) and surgical waste skin tissue (not used for histology) from three healthy subjects undergoing nevus excision were collected to isolate dermal fibroblasts. Peripheral blood samples were drawn from four healthy volunteers and from two MAF donor patients (as identical MHC controls) for the isolation of PBMCs.

### 4.2. Enrichment and Isolation of γδ T Cells

PBMCs from healthy individuals (n = 4) were isolated by using Ficoll–Paque density gradient centrifugation in Leucosep (Greiner Bio-One, Kremsmünster, Austria) tubes according to the instructions of the manufacturer. Next, PBMCs were cultured for 7 days in Roswell Park Memorial Institute (RPMI) 1640 (Gibco™) medium supplemented with 10% fetal bovine serum (FBS) (Gibco™ Thermo Fisher Scientific, Inc., Waltham, MA, USA), 1% penicillin–streptomycin (P/S) (Gibco™, Thermo Fisher Scientific, Waltham, MA, USA), and 1% L-glutamine (Gibco™). For the enrichment of γδ T cells, PBMCs were stimulated with 5 μM ZA (Sigma-Aldrich, St. Louis, MO, USA) and 100 IU/mL interleukin-2 (IL-2; Peprotech, Offenbach, Germany). On the 4th day, the media were changed, and a repeated IL-2 stimulus was added. γδ T cells were isolated through positive selection by using anti-human γδ TCR magnetic microbeads and LS columns (Miltenyi Biotec, Bergisch Gladbach, Germany). The purity of the isolated cell population was confirmed by measuring the ratio of TCR γδ-FITC (Miltenyi Biotec) positive cells/the total number of cells by using flow cytometry (BD FACSCalibur™, BD Biosciences, San Diego, CA, USA). Enrichment and isolation of γδ T cells from PBMCs of two MHC-identical (identical to MHC molecules on their own MAFs) melanoma patients were performed in the same way.

### 4.3. Isolation and Characterization of MAFs

MAFs were isolated from either primary tumors or skin metastases of melanoma patients and characterized as previously described [23,24]. Briefly, the inner tumor mass was minced and digested in 20 mL of DMEM supplemented with 200 IU/mL type IV collagenase and 0.6 IU/mL dispase (Thermo Fisher Scientific). MAFs were separated from melanoma cells by using a differential adhesion/trypsinization method. MAFs’ mesenchymal-stem-cell-like properties and distinctive markers from melanoma cells were characterized previously [23,24]. All MAFs were used in a passage number below eight.

The viability and early apoptosis of MAFs in the presence of different concentrations of ZA were measured by using the CCK-8 assay (Cell Counting kit-8, Dojindo Laboratories, Kumamoto, Japan) and flow cytometry, respectively (please see the details in the Supplementary Methods section).

### 4.4. Apoptosis Assays Using γδ T Cells and MAFs

MAFs were seeded in tissue-cultured and treated 6-well plates (2 × 10^5^ cells/well) for attachment; then, γδ T cells were added in a ratio of 1:1. γδ T cells in co-culture were stimulated with either 1 or 2.5 µM ZA and 100 IU/mL IL-2. As controls, MAFs without γδ T cells and MAFs with unstimulated γδ T cells were used as controls. Cells were co-cultured for 5 days without a change of media. First, the optimal concentration of ZA and co-culture time were determined (Figure 2). Apoptotic populations were analyzed by using flow cytometry with the Cytoflex V5-B5-R3 (Beckman Coulter, Brea, CA, USA) and FlowJo^®^ (Becton Dickinson and Company, Franklin Lakes, NJ, USA) software. The gating strategy consisted of defining the MAF population as CD45− and CD73+ cells and defining the γδ T cell population as CD45+ and CD73− cells. In both populations, early apoptotic (Annexin V+, 7AAD−) and late apoptotic (Annexin V+, 7AAD+) cells were analyzed.

For the apoptosis assay with the optimal conditions, eight subjects’ MAFs and the γδ T cells of healthy donors were used. For an additional apoptosis assay with NDFs, three subjects’ cells and SK-MEL-28 (B-Raf (V600E) mutant and N-Ras wild-type) were used. For the MHC-independence experiments, MAFs and γδ T cells from the same patients (two patients) were used, as they carried identical MHC alleles.

To explore the mechanistic details, we performed further control experiments.

MAFs and γδ T cells were seeded in a ratio of 1:1 in multi-well plates, as above. To demonstrate the role of BTN3A1, a special stimulating anti-BTN3A1 antibody (eBioBT3.1, Thermo Fisher Scientific) clone 20.1 was used at a concentration of 1 µg/mL either alone or with additional stimulation with 1 µM ZA. This particular clone changed the conformation of the BTN3A1 molecule into an active form and enhanced the cytotoxic effect of γδ T cells [52].

To demonstrate the direct involvement of BTN2A1 and γδ TCR, an anti-BTN2A1 (Cat no: HPA019208, Prestige Antibodies^®^, Merck) polyclonal antibody alone or with anti-TCR γδ (Clone B1) (Biolegend, San Diego, CA, USA) was used. IL-2 was added to each condition at a concentration of 100 IU/mL. Anti-BTN2A1 and anti-BTN3A1 antibodies were pre-incubated with MAFs 30 min before adding the ZA-stimulated γδ T cells to the designated wells. Early and late apoptotic populations were measured as described above. These conditions were measured by using n = 3 MAF donors in technical triplicates.

Applied stimuli in each condition and manufacturers’ information can be found in Table 2 and Table 3.

### 4.5. Quantitative RT-PCR Measurements of BTN2A1, BTN3A1, MICA, and MICB Expression

Total RNA was extracted from 5 × 10^5^ fibroblasts (normal dermal fibroblasts and MAFs derived from three patients) or SK-MEL-28 melanoma cells (cat no.: HTB72; purchased from American Type Culture Collection, ATCC; Rockville, MD, USA) using RNeasy mini-kit by Qiagen (Hilden, Germany) according to the manufacturer’s protocol. For cDNA synthesis, M-MLV RT (Moloney-Murine Leukemia Virus Reverse Transcriptase) enzyme (Promega™, Madison, WI, USA) and oligodT primers were used to transcribe 1000 ng of mRNA. To optimize the annealing temperatures of the primers, RT PCR was first run by using the Bioline Immomix Red PCR mix (Meridian Bioscience, London, UK). BTN2A1, BTN3A1, MICA, and MICB transcripts were amplified by using published previously primers [53,54]. PCR products were run on 3% agarose gel.

Subsequently, quantitative PCR was performed with a Roche Lightcycler^®^ 480 thermal-cycler using the Lightcycler^®^ 480 SYBR Green I Master PCR mix (Roche, Basel, Switzerland). A total of 35 cycles were performed with denaturation at 95 °C, at annealing 62 °C, and extension at 72 °C. For the gene expression studies, the melanoma cell line SK-MEL-28 (B-Raf (V600E) mutant and N-Ras wild-type) was used as a positive control. The messenger RNA expression of the housekeeping gene glyceraldehyde 3-phosphate dehydrogenase (GAPDH-∆Ct) was also measured. The ∆∆Ct values were determined by considering the GAPDH mRNA expression of all samples and the target gene mRNA expression of the normal dermal fibroblast sample (∆Ct-control).

### 4.6. Statistical Analysis

The datasets were analyzed by using one-way ANOVA and Dunnett’s post hoc test in the Graphpad Prism 7.0 software. *p*-values of <0.05 were accepted as statistically significant. In the optimization experiments of the apoptosis assay, the correlation between the dose response of the ZA concentrations and the ratio of apoptotic populations was analyzed by using Spearman’s rank correlation test.

## 5. Conclusions

By utilizing our simple in vitro apoptosis model, we demonstrated that γδ T cells with direct ZA activation were capable of inducing apoptosis in MAFs. Based on our study, we provided insights for a better understanding of the tumor microenvironment and, especially, the interaction between anti-tumor γδ T cells and cancer-associated fibroblasts (also known as cancer-associated stromal cells). Our data could be considered in the design of future in vivo experiments or clinical studies to develop new therapeutic approaches to the treatment of melanoma.

## Figures and Tables

**Table 1:** Clinicopathological features of melanoma patients who donated skin samples for isolation of the MAFs used in this study. CM: cutaneous metastasis, DM: distant metastasis, F: female, LNM: lymph node metastasis, M: male, MI: mitosis index, NM: nodular melanoma, PT: primary tumor, SSM: superficial spreading melanoma, wt: wild-type.

Patient. ID	MAF Origin	Gender	Age	Primary Melanoma Details				BRAF	LNM	DM	REF
				Subtype	Breslow (mm)	Clark	MI				
MAF17	PT	F	50	SSM	2.93	IV	14	positive	n/a	yes	[24]
MAF22	PT	M	74	NM	6.23	IV	18	wt	yes	n/a	[24]
MAF31	CM	F	54	unclassifiable	18.21	V	42	wt	yes	yes	[24]
MAF32	PT	F	61	SSM	0.41	II	0	wt	no	no	This study
MAF41	CM	M	43	SSM	0.953	III	4	positive	yes	yes	[24]
MAF43	PT	M	48	unclassifiable	17.5	V	26	wt	no	yes	This study
MAF45	PT	F	90	NM	13.24	IV	46	n/a	n/a	n/a	[24]
MAF47	CM	M	67	SSM	6.18	IV	5	positive	yes	yes	[24]
MAF54	PT	M	74	NM	13.24	V	48	positive	n/a	yes	[24]
MAF55	PT	M	57	unclassifiable	12.3	V	18	positive	yes	yes	[24]
MAF56	CM	F	71	SSM	3.4	IV	12	positive	no	yes	[24]

**Table 2:** Stimuli and antibodies applied in the MAF–γδ T cell co-cultured apoptosis assay. Num-bers represent the bars in Figure 5f. Plus and minus signs refer to their presence or absence in each condition.

	1	2	3	4	5	6	7	8	9	10
IL-2 [100 IU/mL]	(−)	(−)	(+)	(+)	(+)	(+)	(+)	(+)	(+)	(+)
Zoledronic acid [1 μM]	(−)	(−)	(+)	(+)	(+)	(−)	(+)	(+)	(+)	(+)
Anti-BTN3A1 (clone 20.1) [1 μM]	(−)	(−)	(−)	(−)	(−)	(+)	(+)	(−)	(−)	(−)
Anti-BTN2A1 [5 μg/mL]	(−)	(−)	(−)	(−)	(−)	(−)	(−)	(+)	(−)	(+)
Anti-TCR (clone B1) [5 μg/mL]	(−)	(−)	(−)	(−)	(−)	(−)	(−)	(−)	(+)	(+)

**Table 3:** Source of applied stimuli and antibodies in the MAF-γδ T cell co-cultured apoptosis assay.

Antibody	Manufacturer
Mouse IgGκ1 isotype control [P3.6.2.8.1]	eBioscience™, Thermo Fisher Scientific, Waltham, MA, USA
Anti-TCRγ/δ antibody (Clone B1)	Biolegend^®^, San Diego, CA, USA
Anti-BTN3A1 monoclonal antibody (eBioBT3.1 (20.1))	eBioscience™, Thermo Fisher Scientific, Waltham, MA, USA)
Anti-BTN2A1 Polyclonal antibody	Prestige Antibodies^®^ Merck, Darmstadt, Germany)
Rabbit IgG isotype control	Southern Biotech, Birmingham, AL, USA)

## Data Availability

All data generated or analyzed during this study are included in this published article. Further data are available upon reasonable request from the corresponding author.

## Supplementary Materials

The following supporting information can be downloaded at: https://www.mdpi.com/article/10.3390/ijms241612893/s1. Reference [55] is cited in the Supplementary Materials.
