# Supplementary material for: Targeting Melanoma-Associated Fibroblasts (MAFs) with Activated γδ (Vδ2) T Cells: An In Vitro Cytotoxicity Model
Source: Int J Mol Sci. 2023 Aug 17;24(16):12893. doi: 10.3390/ijms241612893 (PMC10454423; doi:10.3390/ijms241612893)
Supplement: Supplementary file 1 [file ijms-24-12893-s001.zip › ijms-2517666-supplementary.pdf]

Supplementary Methods

ZA Cell Counting Kit-8 (CCK-8) assay on MAFs

MAFs from 3 donors were plated in triplicate in 96-well plates (25,000 cells/well) in DMEM supplemented with 20% FBS, P/S, and L-glutamine. On the next day, the medium was changed to mimic the co-culture environment (RPMI, 10% FBS, P/S, L-glutamine, and 100 IU/mL IL-2), and the cells were incubated with 0–5 μMolar ZA for 5 days.

For the CCK-8 assay (Cell Counting Kit-8, Dojindo Laboratories, Kumamoto, Japan), the medium was changed to 100 μL/well of serum-free RPMI, P/S, and L-glutamine, and 10 μL of CCK-8 reagent was added. Cells were incubated in a cell culture incubator (37 °C, 5% CO_2) for an hour, and the OD at 450–630 nm was measured in an ELISA plate reader. Empty wells (no cells, only medium) were used as blank controls.

Flow cytometry apoptosis assays in MAFs

MAFs from 3 donors were plated in 12-well plates in RPMI medium supplemented with 10% FBS, P/S, and L-glutamine, as well as 0–5 μM ZA. Cells were incubated for 5 days without a change of media. Apoptotic populations were analyzed by using flow cytometry, as described in the Methods section (*4.4 Apoptosis assays using γδ T cells and MAFs).*

Flow cytometry apoptosis assays in normal dermal fibroblasts (NDFs) and the SK-MEL-28 cell line

NDFs from 3 donors and the SK-MEL-28 melanoma cell line were plated in 12-well plates in RPMI medium supplemented with 10% FBS, P/S, and L-glutamine, as well as 100 IU/mL IL-2 and 1–2.5 μM ZA. Cells were incubated for 5 days without a change of media. Apoptotic populations were analyzed by using flow cytometry, as described in the Methods section (*4.4 Apoptosis assays using γδ T cells and MAFs).* SK-MEL-28 cells were gated as CD45- and CD73+ [55]. For the statistical analysis, two-way ANOVA was performed with a Bonferroni post-hoc test.

BTN3A1 surface expression on NDFs, MAFs, and the SK-MEL-28 cell line

The surface expression of BTN3A1 was analyzed on NDFs, MAFs, and the SK-MEL-28 cell line by using flow cytometry with Cytoflex V5-B5-R3 (Beckman Coulter) and the FlowJo® (Becton Dickinson and Company) software. Briefly, cells were labeled with a PE-conjugated anti-BTN3A1 (CD277) antibody (Miltenyi Biotec). The gating strategy consisted of cell size differentiation, and BTN3A1-positive cells were determined by using unstained controls of each cell type.

Statistical analysis

The datasets of the apoptosis assay with NDFs and the SK-MEL-28 cell line were analyzed by using two-way ANOVA and Bonferroni's post-hoc test with the Graphpad Prism 7.0 software. The BTN3A1 expression data were analyzed by using one-way ANOVA and Tukey's post-hoc test. *p*-values of <0.05 were accepted as statistically significant.

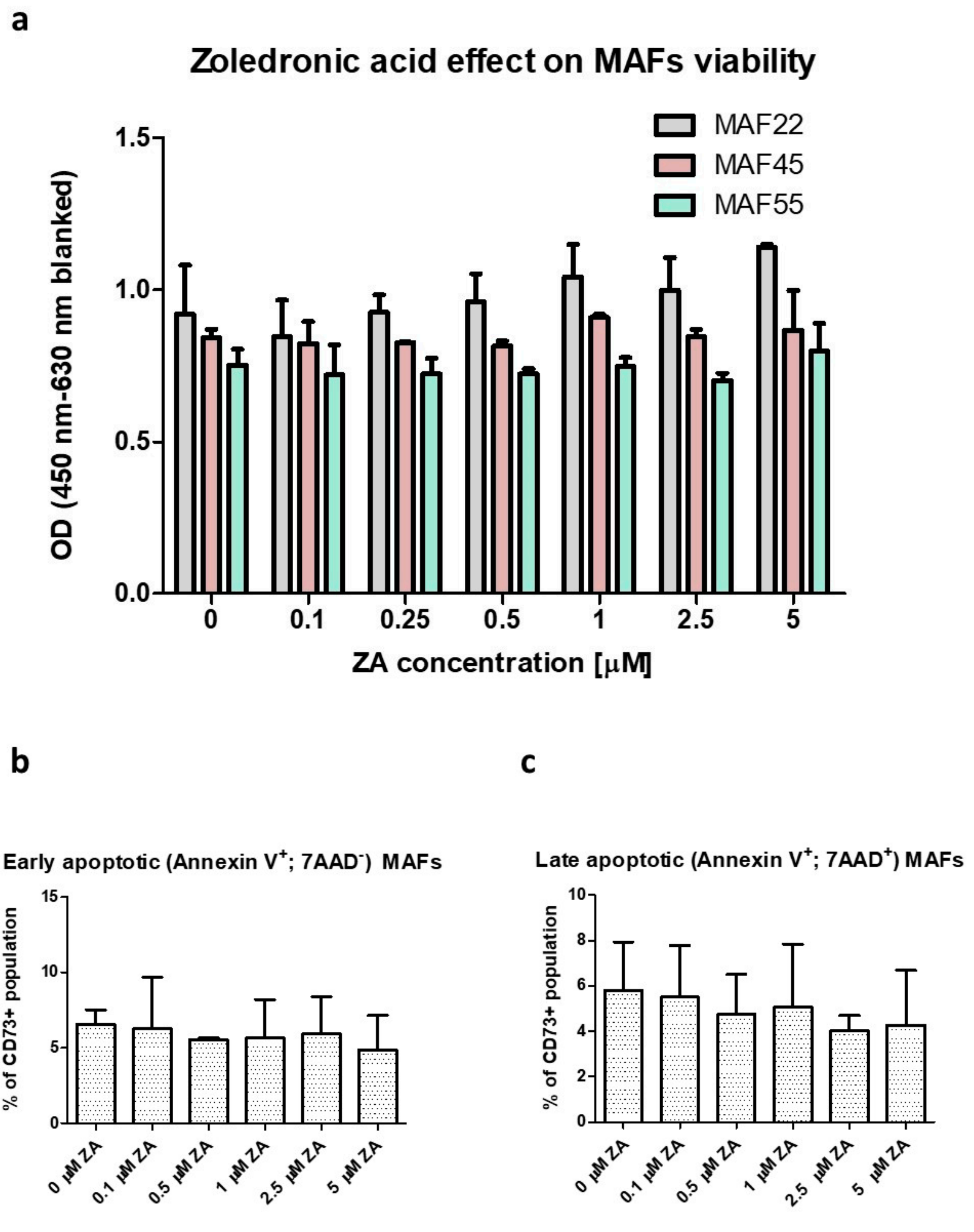

Figure S1. Effect of ZA on the viability of MAFs. (a) OD values measured in the CCK-8 assay for ZA (0–5 μM)-treated MAFs (n=3). Non-treated MAFs were used as controls. (b) Percentages of early apoptotic (Annexin V+, 7AAD-) and (c) late apoptotic (Annexin V+, 7AAD+) populations of MAFs [CD73+; CD45-] (n=3). Control: MAFs without ZA treatment. Error bars represent means ± SD.

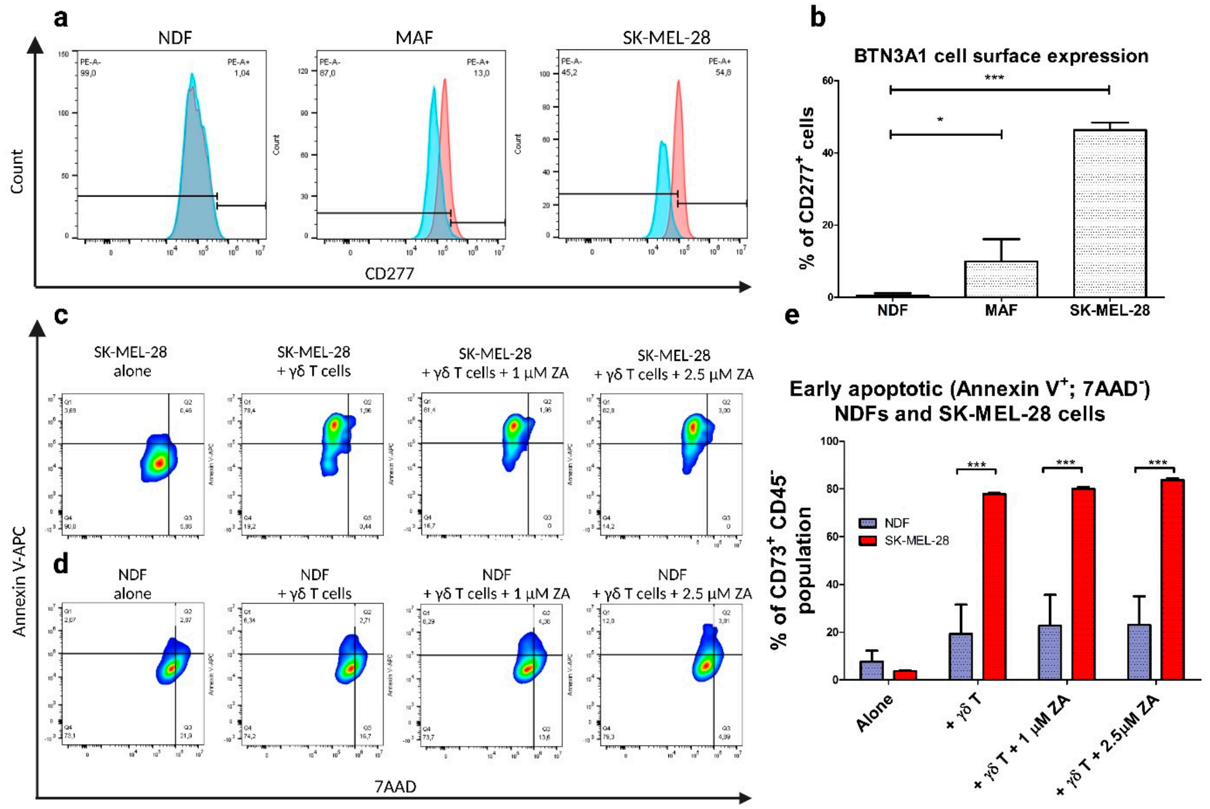

Figure S2. The surface expression of BTN3A1 and γδ-T-cell-induced apoptosis in fibroblasts and SK-MEL-28 cells. (a) Representative histograms of BTN3A1 expression in NDFs, MAFs, and SK-MEL-28 cells. (b) BTN3A1 expression on fibroblasts and the SK-MEL-28 cell line. (c) Representative density plots of early apoptotic (Annexin V+; 7AAD-) populations in the SK-MEL-28 cell line. (d) Representative density plots of early apoptotic (Annexin V+; 7AAD-) populations in NDFs. (e) Percentage of early apoptotic populations of NDFs and the SK-MEL-28 cell line [CD73+ CD45-] after 5 days of co-culture with γδ T cells. Control: co-culture with unstimulated γδ T cells. (d) Representative histograms of BTN3A1 expression in NDFs (n=3), MAFs (n=3), and the SK-MEL-28 cell line. Error bars represent means ± SD .*$p < 0.05$ **$p < 0.01$ and ***$p < 0.001$.
